# Contact Lens Solution–associated *Acanthamoeba* and *Fusarium* Keratitis

**DOI:** 10.3201/eid1609.091381

**Published:** 2010-09

**Authors:** John D. Bullock, Ronald E. Warwar

**Affiliations:** Author affiliations: Wright State University Boonshoft School of Medicine, Dayton, Ohio, USA

**Keywords:** Acanthamoeba keratitis, Fusarium keratitis, contact lens solutions, drug contamination, ameba, fungus, letter

**To the Editor:** Verani et al. (*1*) detailed the 2004–2007 outbreak of *Acanthamoeba* keratitis (AK) in persons wearing soft contact lenses who used Complete MoisturePlus (CMP) multipurpose contact lens solution (Advanced Medical Optics, Santa Ana, CA, USA). They noted similarities between the AK outbreak and the *Fusarium* keratitis (FK) outbreak of 2004–2006, including the concomitant time frame and association with a particular solution, ReNu with MoistureLoc (Bausch & Lomb, Rochester, NY, USA). Both solutions were new products introduced within 1 year before the respective outbreaks.

In neither outbreak was the solution contaminated; in both outbreaks, implicated bottles were from multiple lots, suggesting that each outbreak resulted from insufficient antimicrobial activity. However, in the FK outbreak, all reported cases involved bottles produced at 1 (Greenville, SC, USA) of 4 multinational Bausch & Lomb manufacturing plants (*2*). After a Food and Drug Administration inspection of the Greenville facility, Bausch & Lomb was cited for inadequacies in temperature control during production, storage, and transport of its products in and beyond the plant (*3*).

We experimentally demonstrated that, when exposed to prolonged temperature elevation, ReNu with MoistureLoc loses more in vitro fungistatic activity than do other contact lens solutions. We concluded that improper temperature control of ReNu with MoistureLoc may have contributed to the FK outbreak (*4*). We are aware of no other theory that adequately explains why only ReNu with MoistureLoc from only 1 plant was implicated.

CMP was manufactured and used internationally; AK has a much higher incidence in Europe and Hong Kong than in the United States (*5*), and CMP–associated AK has been reported internationally (*6*). Therefore, it would seem critical to know, and we would like the authors to comment on, whether the geographic pattern of the AK coincided with distribution of CMP solution from >1 Advanced Medical Optics manufacturing plants and, if so, the relevance of that information.
