# Supplementary material for: Contact Lens Solution–associated Acanthamoeba and Fusarium Keratitis
Source: Emerg Infect Dis. 2010 Sep;16(9):1501–3. doi: 10.3201/eid1609.091381 (PMC3294962; doi:10.3201/eid1609.091381)
Supplement: Related texts

# Reply: Contact Lens Solution–associated *Acanthamoeba* and *Fusarium* Keratitis

**DOI:** 10.3201/eid1609.100933

**Authors:** Jennifer R. Verani, Jonathan S. Yoder, Sharon L. Roy

**Affiliations:** Author affiliation: Centers for Disease Control and Prevention, Atlanta, Georgia, USA

**In Response:** We thank Bullock and Warwar for offering their theory of potential consequences of manufacturing inadequacies in temperature control during production of ReNu with MoistureLoc (Bausch & Lomb, Rochester, NY, USA) associated with the *Fusarium* keratitis (FK) outbreak during 2004–2006 (*1*). They note the substantial similarities between the FK outbreak and the *Ancanthamoeba* keratitis (AK) outbreak that we reported (*2*). They inquire whether the geographic pattern of AK outbreak–associated cases coincides with distribution of >1 manufacturing plants for the associated product, Complete MoisturePlus (CMP) multipurpose contact lens solution (Advanced Medical Optics [AMO], Santa Ana, CA, USA).

We obtained lot numbers for 22 bottles of CMP that AK case-patients used before symptom onset. Because no lot number was repeated, intrinsic contamination was unlikely as the source of the AK outbreak; the geographic and temporal distribution of cases further argued against a point-source outbreak. All 17 lot numbers for which AMO plant of origin was determined were manufactured in Spain (Food and Drug Administration, pers. comm.). According to a press release from AMO in November 2006, the “vast majority of AMO's contact lens solution products distributed in the U.S.” were manufactured in the company’s production facility in Spain, 1 of its 2 international manufacturing plants (*3*).

CMP was produced and used internationally at the time of the US multistate outbreak (*4*). Por and colleagues (*5*) reported an increase in the number of AK cases among contact lens users in Singapore that temporally coincided with the US outbreak. However, their retrospective case series did not include a control group; therefore, measuring associations between particular contact lens products and AK was not possible for those case-patients. The authors reported that a case–control study was underway, and we look forward to seeing the results of that investigation to better understand the magnitude of AK cases associated with CMP use.

## References

[1] Bullock JD, Warwar RE. Contact lens solution–associated *Acanthamoeba* and *Fusarium* keratitis [letter]. Emerg Infect Dis. 2010;16:1501–2. 10.3201/eid1609.09138120735950PMC3294962

[2] Verani JR, Lorick SA, Yoder JS, Beach MJ, Braden CR, Roberts JM, National outbreak of *Acanthamoeba* keratitis associated with use of a contact lens solution, United States. Emerg Infect Dis. 2009;15:1236–42. 10.3201/eid1508.09022519751585PMC2815976

[3] US Food and Drug Administration. Advanced Medical Optics announces voluntary recall of 18 lots of Complete(R) MoisturePLUS(TM) contact lens care products distributed and sold in the U.S. Includes certain lots of 12-ounce bottles and active packs [cited 2010 Jun 11]. http://www.fda.gov/Safety/Recalls/ArchiveRecalls/2006/ucm112073.htm

[4] US Food and Drug Administration. Advanced Medical Optics, Inc. COMPLETE® MoisturePLUS™ multi-purpose contact lens solution [2010 Jun 11]. http://www.fda.gov/MedicalDevices/Safety/RecallsCorrectionsRemovals/ListofRecalls/ucm062478.htm

[5] Por YM, Mehta JS, Chua JL, Koh TH, Khor WB, Fong AC, *Acanthamoeba* keratitis associated with contact lens wear in Singapore. Am J Ophthalmol. 2009;148:7–12.e2.10.1016/j.ajo.2009.02.03019403109
